# Identification of novel functional sites in the Ca_v_1.3 calcium channel α1-subunit using evolutionary modeling

**DOI:** 10.1073/pnas.2602636123

**Published:** 2026-08-03

**Authors:** Xuechen Tang, Horia C. Hermenean, Alesia Yakimchyk, Petronel Tuluc, Nadine J. Ortner, Klaus R. Liedl

**Affiliations:** ^a^ Department of Theoretical Chemistry, University of Innsbruck, Innsbruck 6020, Austria; ^b^ Department of Pharmacology and Toxicology, University of Innsbruck, 6020 Innsbruck, Austria; ^c^ Gottfried Schatz Research Center, Division of Medical Physics and Biophysics, Medical University of Graz, 8010 Graz, Austria

**Keywords:** voltage-gated calcium channels, evolutionary modeling, *CACNA1D*, channelopathy, electrophysiology

## Abstract

Voltage-gated calcium channels (VGCCs) regulate differentiation, function, and survival of excitable cells, and pathogenic variants cause diverse disorders. Most known disease-associated VGCC mutations affect well-characterized regions controlling voltage-dependent gating and channel kinetics, while many residues remain functionally unannotated. We developed an evolutionary model to predict the pathogenic potential of residues in the pore-forming Ca_v_1.3 subunit, previously validated with de novo gain-of-function variants linked to neurodevelopmental diseases. Here we show that the model recapitulates established functional regions and prospectively identifies functional sites at single–amino acid resolution. Electrophysiological analyses of five predicted variants across multiple channel domains confirmed functional alterations. The approach also captures loss-of-function variants typically pathogenic only in the homozygous state, establishing a predictive framework for identifying and functionally characterizing pathogenic variants in Ca_v_1.3 and related ion channels.

## Significance

Voltage-gated calcium channels regulate critical physiological processes, and some genetic variants in *CACNA1D* can cause neurodevelopmental and cardiac disorders. We demonstrate that evolutionary modeling based on sequence covariation can predict functionally important channel sites at single amino acid resolution. Our algorithm correctly identified known functional regions and revealed previously uncharacterized sites, which were validated through electrophysiological experiments showing diverse gating changes ranging from nonconducting channels to complex gain-of-function alterations. Importantly, the model captures both gain-of-function variants linked to neurodevelopmental disease and loss-of-function variants causing deafness and cardiac dysfunction. By enabling rapid identification of disease-causing variants and their functional consequences, this framework provides a crucial foundation for understanding the molecular origins of channelopathies and advancing personalized therapeutic strategies.

Voltage-gated calcium channels (VGCCs) are essential regulators of intracellular calcium homeostasis controlling a wide range of cellular functions, including electrical activity, neurotransmitter release, muscle contraction, hormone secretion, and gene expression (1, 2). Consequently, changes of channel gating can disrupt electrochemical signaling causing various human disorders referred to as calcium channelopathies (3). Functionally, pathogenic genetic variants can display clear loss- or gain-of-function or can have complex mixed features (3), resulting in altered calcium signaling and pathophysiological conditions.

VGCCs belong to the superfamily of voltage-gated ion channels that show a high degree of sequence homology (InterPro accession: IPR005821) and share many gating mechanisms (4, 5). Numerous key functional features and residues have been identified by analysis of amino acid properties (6), mutagenesis, electrophysiology, X-ray crystallography, and cryo-EM structures (7, 8). Besides these experimental techniques, a completely independent and orthogonal way of looking at channel function is to evaluate evolutionary data. One way to exploit this information is to infer interactions between residues using sequence covariation, based on the fact that functionally coupled residues would coevolve to maintain the fold over time (9–12). This sequence covariation can be used to deduce context-dependent conservation and predict the pathogenicity of genetic variations (13). We recently published an evolutionary model for the *CACNA1D* gene, which encodes the pore-forming α1-subunit of the L-type Ca_v_1.3 VGCC, that showed good predictability of disease risk for known pathogenic variants in different channel regions (14). Previously, we and others have identified and functionally characterized many key residues found to be mutated in patients with Ca_v_1.3 channelopathies (15–23). However, due to advances in genome sequencing techniques, the number of disease-associated genetic variants is rapidly increasing, making the functional characterization a very challenging and tedious task.

While the previous study established the predictive performance of the model for known pathogenic variants, the present work extends this approach by prospectively identifying previously uncharacterized functional sites across the channel and experimentally validating their impact. To achieve this objective, here we used a combination of evolutionary prediction, electrophysiological functional validation, and structural analysis. Importantly, these approaches serve complementary roles: evolutionary modeling provides a predictive, structure-independent framework to identify residues with high functional sensitivity, whereas structural modeling is primarily used to mechanistically interpret experimentally observed effects. In contrast to structural approaches, which are typically limited in their ability to predict functional relevance a priori in large and dynamic membrane proteins, the evolutionary model enables unbiased identification of candidate functional sites across the entire channel. To identify residues beyond classically known Ca_v_1.3 α1-subunit functional sites we used our recently established evolutionary model for the prospective prediction of the pathogenicity of Ca_v_1.3 amino acid substitutions (14). Mapping the obtained pathogenicity scores on the channel structure revealed channel parts and residues previously unrecognized as potential functional modifiers. Retrospective calcium current recordings of selected mutations confirm their pathogenic potential while structural analysis provides the mechanistic interpretation. Importantly, we can also show that our algorithm detects mutations inferring a wide range of functional changes, from complete loss-of-function to more complex alterations combining gain- and loss-of-function features. We thereby demonstrate that the combination of orthogonal evolutionary prediction, functional validation, and structural interpretation represents a powerful toolbox. This toolbox helps to identify the amino acids relevant for ion channel function and to allow rapid assessment of the pathogenic potential of newly identified patient-derived *CACNA1D* variants, crucial for diagnosis as well as tailored care and therapeutic approaches.

## Results

### Evolutionary Predictions Match Known Functional Sites.

**Fig. 1.**
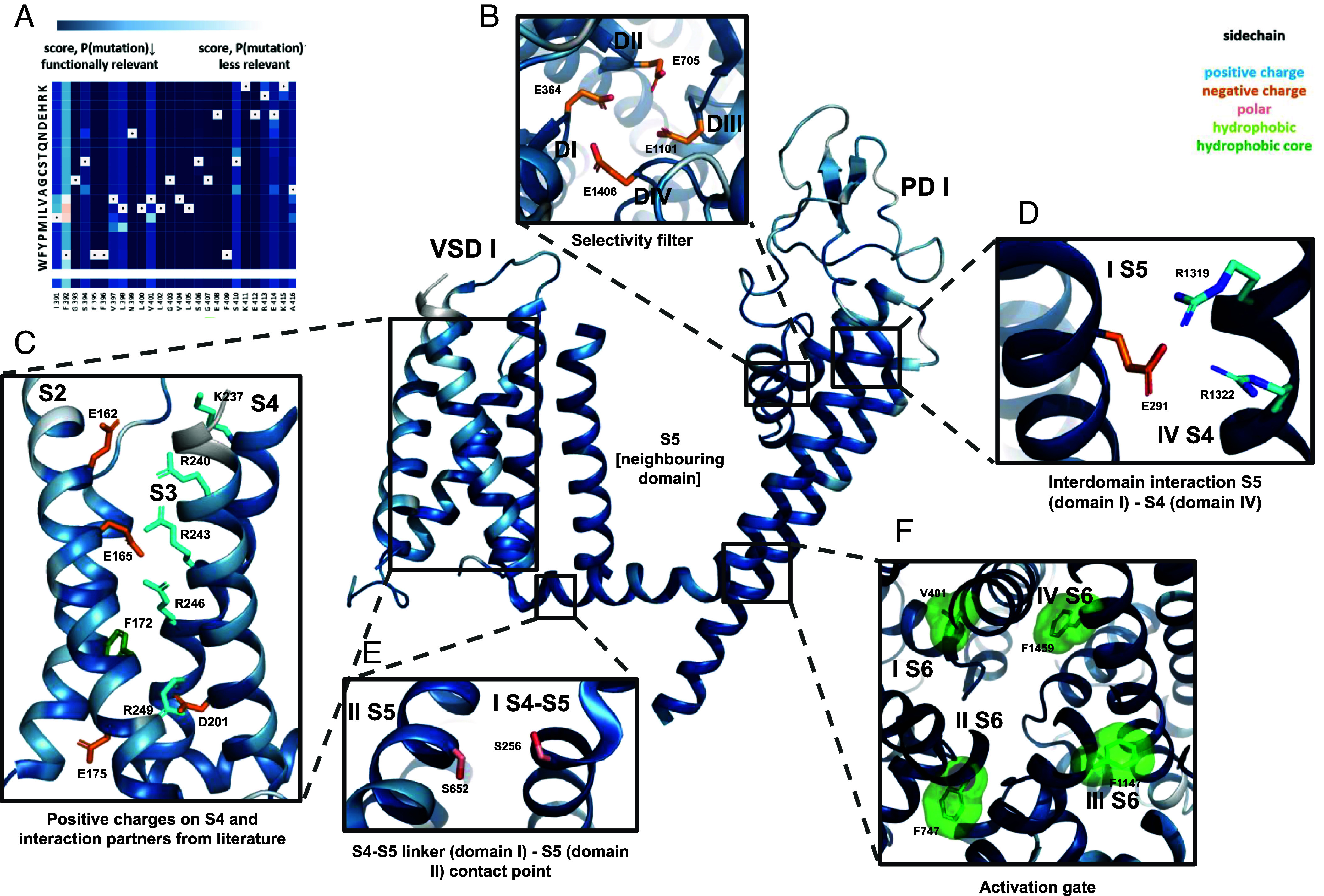
Evolutionary pathogenicity predictions overlap with established functional and structural sites of Ca_v_1.3. Cartoon representation of domain I of the Ca_v_1.3 α1-subunit, comprising voltage-sensing domain I (VSD I) and pore domain I (PD I). Zoomed regions highlight residues with known functional relevance: (*A*) epistatic score matrix, where darker blue indicates lower scores and higher predicted pathogenicity, lighter blue to orange indicates higher scores and lower pathogenicity, and white with a central dot denotes wild-type (WT); (*B*) residues forming the structural basis for ion selectivity; (*C*) residues of the charge transfer center; (*D* and *E*) residues mediating interdomain interactions between S4 and S5, and between the S4–S5 linker and S5; and (*F*) residues involved in hydrophobic gating. Backbone coloring reflects evolutionary algorithm–derived pathogenicity scores shown in (*A*), with darker colors indicating more negative scores, higher context-dependent conservation, and increased likelihood of deleterious substitutions. For visualization, residues with scores above −4.6 (low predicted pathogenicity) are shown in white. Side chains are colored by physicochemical class: polar (salmon), negative (orange), positive (cyan), and hydrophobic (green). Residue numbering differs from EU363339 due to inclusion of exons 32 and 44: E1406 to E1391, F1459 to F1444, R1319 to R1304, R1322 to R1307.

Recently, we established an algorithm based purely on evolutionary data that correctly predicted the pathogenicity of genetic variants linked to neurodevelopmental and endocrine disorders caused by activity-enhancing channel gating changes (14). To exploit this model for the study of the vast majority of amino acids in the Ca_v_1.3 α1-subunit, first we validated its predictive power for sites and residues with well-described known functional roles. This is illustrated in Fig. 1 on the example of domain I of the four homologous channel repeats. The epistatic scores (i.e., tolerability for mutagenesis) were calculated for the potential substitutions with all amino acids (Fig. 1*A*) and presented on a color saturation scale overlaid on channel structure. The darker blue represents a lower score and therefore a higher pathogenicity potential (Fig. 1*A*). Each domain consists of the voltage sensing domain (VSD) comprising of four (S1 to S4) transmembrane segments coupled via the S4–S5 linker to the pore domain (PD) built by S5, S6, and its connecting loops. Voltage sensing and channel gating is triggered by the outward translocation of the S4 helix upon membrane depolarization mechanisms (5, 24, 25). This conformational change is caused by electrostatic forces acting on the S4 positively charged amino acids and aided by sequential H-bond interactions with negative countercharges in the other segments of the same VSD (Fig. 1*C*) (24–27) or a neighboring VSD (28). In addition, the voltage sensor must be sealed against permeating ions by conserved phenylalanines in each S2 helix (F172 in Fig. 1*C*) to form a hydrophobic constriction site (HCS) (29, 30). Indeed, our model predicts low tolerability for substitution of these crucial residues, while less specific hydrophobic residues facing the membrane are mostly predicted to be less conserved and less harmful when mutated (light color; Fig. 1*C*). Consistent with these findings, also known functional sites in the PD were correctly identified by our model as clusters of residues predicted to be highly pathogenic upon mutation. This includes the calcium ion-coordinating glutamates in the selectivity filter (Fig. 1*B*), and the ring of hydrophobic residues in the lower PD part (“activation gate,” Fig. 1*F*) of which two, V401 and F747, have been found mutated in patients with neurodevelopmental diseases and impact channel function (15, 31–33). Besides individual residues, the algorithm also identified known interdomain interaction sites. One of those, S652 in the S5 of domain II, was predicted to be pathogenic when mutated, as was its interaction partner S256 in the S4–S5 linker of domain I (18) (Fig. 1*E*). Indeed, also the S652L substitution was shown to significantly alter Ca_v_1.3 channel gating and is associated with neurodevelopmental and endocrine dysfunction (15, 18, 34). Similarly, another interdomain interaction site between voltage sensor IV and E291 on S5 of domain I via polar contacts (Fig. 1*D*) is also predicted to be highly pathogenic, confirmed by electrophysiological experiments (28). Together, we can establish that our evolutionary model (14) correctly predicts the low tolerance of functionally crucial amino acids for mutagenesis (Fig. 1).

### Clusters of Predicted Pathogenic Residues Outside the Classical Functional Sites.

**Fig. 2.**
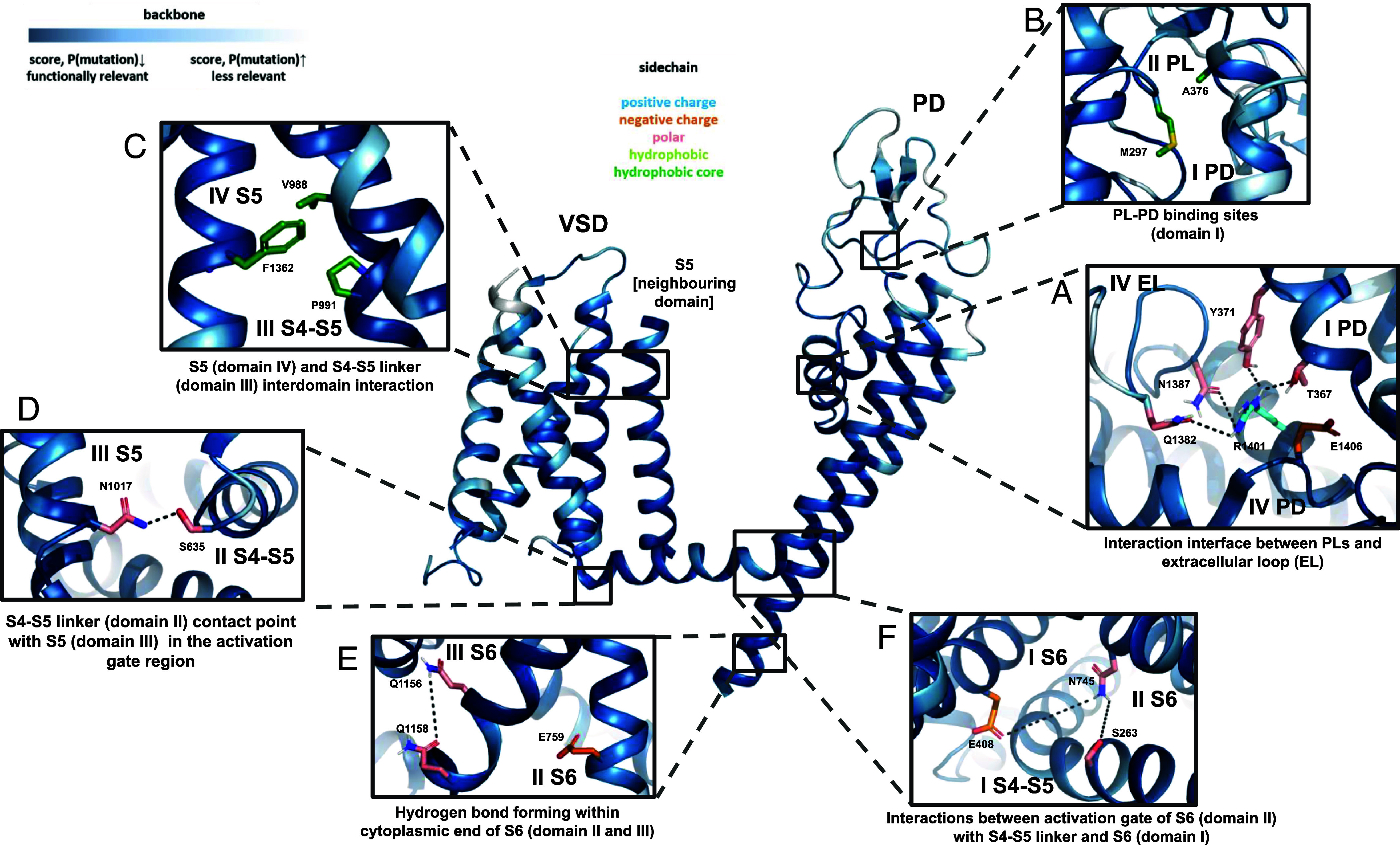
Pathogenicity predictions based on our evolutionary model outside the classical functional sites. Cartoon illustration of VSD and PD of the Ca_v_1.3 α1-subunit. Residues are highlighted in zoom (boxes): (*A*) R1401 participates in multiple polar contacts between PLs (I and IV) and the extracellular loop (IV); (*B*) A376 connects pore loop (PL) to the rest of the pore domain (PD); (*C*) F1362 mediates the coupling in the S4–S5 interface (between IV S5 and III S4); (*D*) S635 establishes connection between II S4–S5 linker and III S5 in the activation gate region; (*E*) Q1156 determines the correct movement of III S6 relative to II S6 in response to the change in the channel state; (*F*) N745 participates in the interaction network surrounding the AG region. The color of the backbone represents the score obtained using the same evolutionary based algorithm as in Fig. 1. The side chains are colored according to the type of residues shown in Fig. 1.

Next, we used our validated model to identify previously unknown or less studied sites involved in Ca_v_1.3 α1 channel gating. Notably, clusters of residues predicted to be highly pathogenic if mutated surround the well-studied functional sites in the PD (Figs. 1 and 2). For the extracellular part of PD, the predicted pathogenic cluster extends beyond the selectivity filter glutamates and the classical conduction loop. It reaches several contact points between pore loops as well as those connecting the pore loop to the rest of the PD (Fig. 2*A*). Despite its position outside the ion path, this finding is consistent with the distribution of mutations with high actual pathogenic impact in ion channels (35). Within the lower PD, residues of the activation gate with small ion path radius and proven functional and/or clinical pathogenicity were among the sites predicted to be most conserved (Fig. 1). Along the S6 helices containing these sites, not only the classical LAIA motif in domain II (36) and its analogues in other domains, but also less studied residues above and below this hotspot were predicted to be highly pathogenic (Fig. 2*B*). Mutagenesis studies (37–39) and the presence of pathogenic variants in voltage-gated sodium channels (https://scn-portal.broadinstitute.org/) support this extended distribution of functionally relevant residues beyond the classical functional core. Among the residues predicted to be most relevant are homologs of the inner gate identified by cryoEM in low voltage-activated T-type Cav3 channels (40). These residues were not classified as gatekeepers in L-type Cav1 VGCCs. Since the sequence ensemble on which our model is based includes only Cav1 isoforms, but not Cav3, the predicted importance of those residues must derive from their functional role in Cav1 channels. This suggests that this ring may contribute to channel gating through mechanisms that are at least partially distinct from the canonical activation gate (Fig. 1*F*), although further experimental validation will be required to fully establish this relationship.

The connecting residues between the PD and the VSD also display high predicted pathogenicity upon mutation (Fig. 2*D*). On the S4–S5 linker, sites facing the activation gate are highly conserved (Figs. 1 and 2). In particular, both the hydrophobic residues, which are thought to be important for preventing pore leakage, and polar residues show high predicted pathogenicity. Furthermore, most neutral residues on S4 appear intolerable to substitution particularly those in close contact with S5 (Fig. 2*C*).

In contrast to the large clusters of predicted important residues in the PD, the voltage sensors carry much fewer predicted pathogenic sites besides the classic S4 gating charges and a number of polar and hydrophobic residues highlighted above (Figs. 1*C* and 2*A*). This is in line with the high prevalence of disease-associated mutations in the PD, which is consistent with a previous study showing the highest enrichment of pathogenic ion channel variants within and surrounding the central pore (35). Therefore, our evolution-based model can faithfully predict pathogenic residues and functional sites along the entire Ca_v_1.3 protein and potentially predict many novel amino acid interactions.

### Diverse Functional Impact of Selected Variants from Newly Identified Clusters.

To validate this orthogonal approach and add mechanistic and functional insights, we used whole-cell patch-clamp recordings in transiently transfected tsA201 cells and structural modeling. For that, we selected five variants predicted to be pathogenic [epistatic score < −4.6 (14)] and located at different channel regions for which no functional role has been shown in Ca_v_1.3 or other ion channels. These variants were primarily selected based on evolutionary predictions to probe functional relevance across different channel regions and do not necessarily correspond to clinically identified patient variants. Moreover, to date, we have focused exclusively on de novo (i.e., heterozygous) variants linked to neurodevelopmental and/or endocrine diseases and enhanced channel activity (15). Interestingly, also the putative loss-of-function A376V variant, associated with sinoatrial node and deafness (SANDD) syndrome when homozygous (41, 42), showed an epistatic score of −4.67 indicative of pathogenicity. Thus, we also included this variant to broaden our approach and demonstrate that our framework is capable of detecting not only high-impact pathogenic gain-of-function variants but also loss-of-function variants that manifest disease only in the homozygous state.

**Fig. 3.**
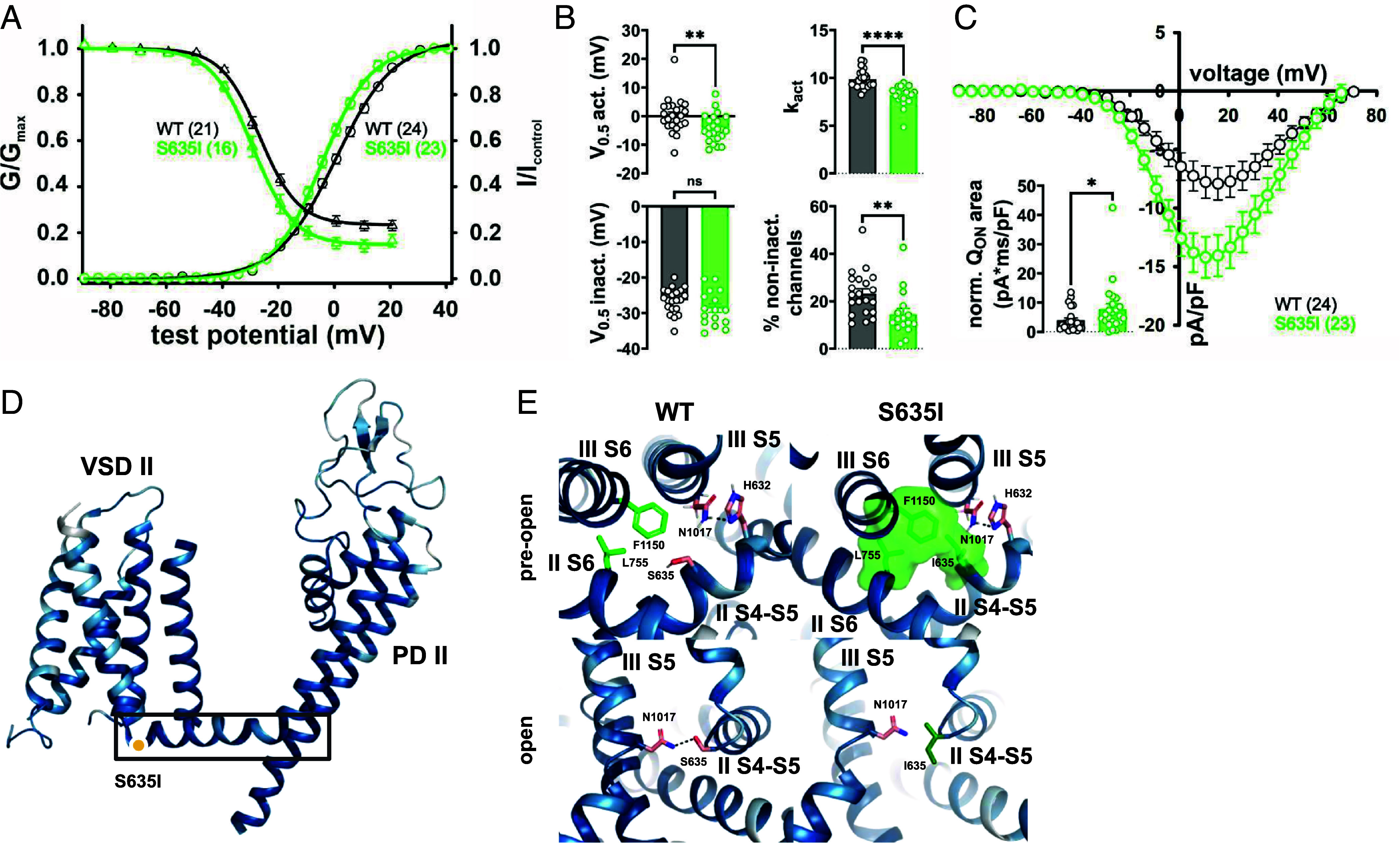
Structural and functional consequences of the S635I variant (domain II, S4–S5 linker). (*A*–*C*) shows that S635I induces a leftward shift of activation (WT: n = 24; S635I: n = 23), enhanced steady-state inactivation (WT: n = 21; S635I: n = 16) and increased current density (WT: n = 24; S635I: n = 23). (*D*) shows the position of S635I in domain II. The structural illustration of the mutation effect in (*E*) shows that S635I establishes an additional hydrophobic contact with the activation gate region at the bottom of the II S6 helix in the preopen state, facilitating channel opening at lower voltage. In the open state, mutation to apolar isoleucine (in green) disrupts a stabilizing polar contact between S635 (in salmon) and N1017 in III S5, facilitating transition to the inactivated state. The side chains are colored according to the type of residues shown in Fig. 1.

Calcium current recordings show that the S635I mutation, located on the S4–S5 linker of domain II (Fig. 3*D*), causes a significant shift by ~−4.5 mV in the voltage dependence of activation toward more hyperpolarized membrane potentials (Fig. 3 *A* and *B* and *SI Appendix*, Table S1). Additionally, the steeper slope of voltage dependence of activation indicates a better coupling of voltage sensing to channel activation (Fig. 3 *A* and *B* and *SI Appendix*, Table S1). Current densities were significantly increased, possibly due to increased surface channel expression evident as increased in gating currents, Q_ON_ (Fig. 3*C* and *SI Appendix*, Table S1). While the voltage dependence of inactivation was unaltered, S635I channels showed a lower fraction of noninactivated channels compared to WT (Fig. 3 *A* and *B* and *SI Appendix*, Table S1). Despite that, the inactivation and deactivation kinetics were not affected (*SI Appendix*, Fig. S2 and Table S2). Structural analysis shows that isoleucine 635 can establish an additional hydrophobic contact with the activation gate region at the bottom of the S6 helices in the preopen state (Fig. 3 *E*, *Top*) which is not established by serine 635. Consistent with electrophysiological data, this could explain the channel opening at lower voltage as this interaction could increase the coupling between the VSD and the gate. In the open state, mutation to an apolar isoleucine (in green) disrupts a stabilizing polar contact between S635 (in salmon) and N1017, which might destabilize the open state and facilitate the transition to the inactivated state (Fig. 3 *E*, *Bottom*).

**Fig. 4.**
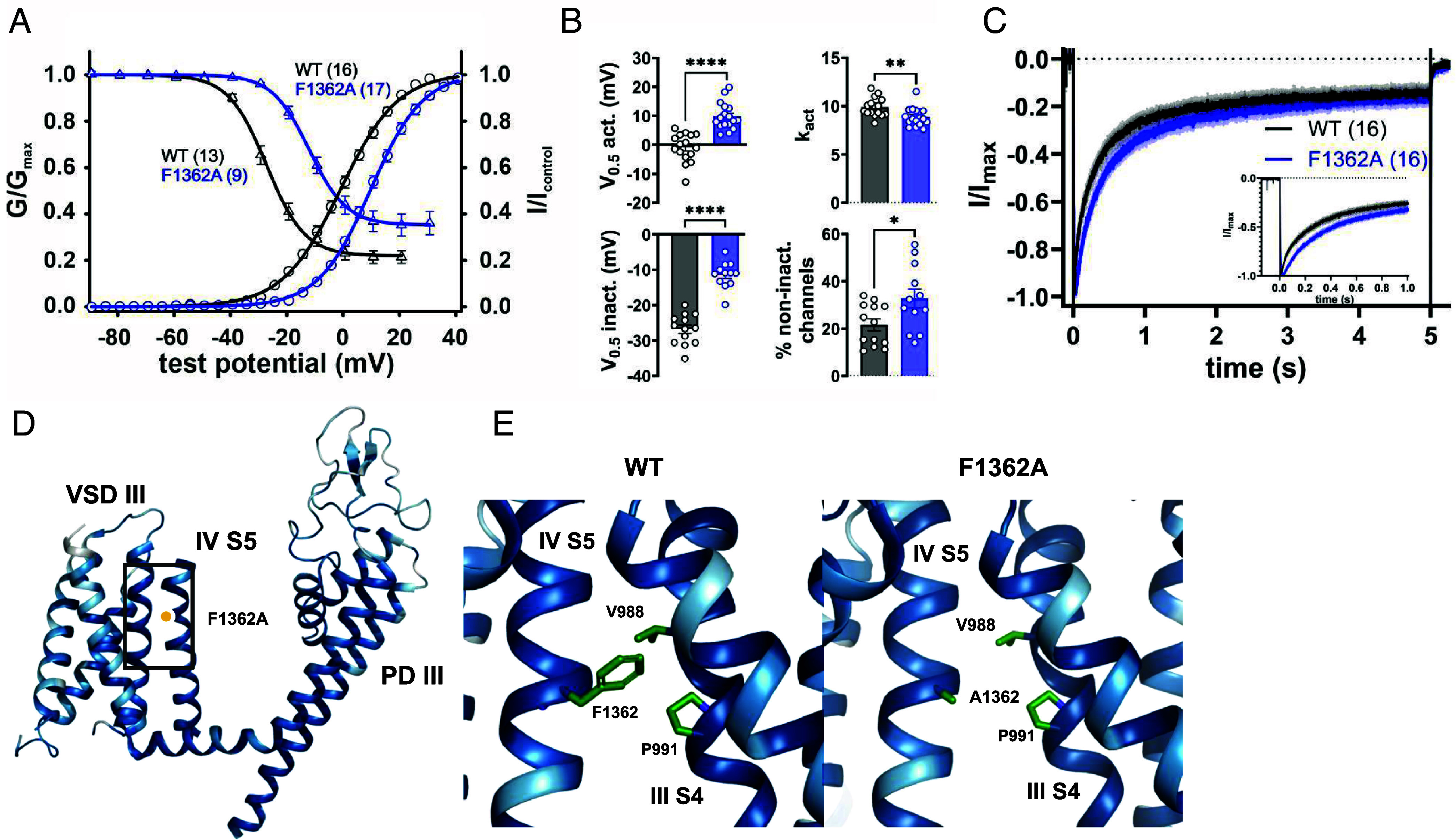
Structural and functional consequences of the F1362A variant (domain IV, S5). (*A*–*C*) The F1362A mutation induces a rightward shift in the voltage-dependent activation (WT: n = 16; F1362A: n = 17) and steady-state inactivation (WT: n = 13; F1362A: n = 9) together with slowed inactivation (WT: n = 16; F1362A: n = 16). (*D*) marks the position of F1362A in the S4–S5 interface. Structural illustration of the mutation effect in (*E*) shows that F1362A loosens the coupling in the S4–S5 interface by disrupting the hydrophobic and polarizable contacts. The side chains are colored according to the type of residues shown in Fig. 1.

The F1362 residue is located in the S5 helix of domain IV (Fig. 4*D*). Structural modeling suggests that the F1362A substitution loosens the coupling in the S4–S5 interface by disrupting the hydrophobic and polarizable contacts (Fig. 4*E*). Consistent with this hypothesis, F1362A mutation causes an ~+11 mV shift of the voltage dependence of activation and ~+15 mV shift of the voltage dependence of inactivation toward higher membrane potentials (Fig. 4 *A* and *B* and *SI Appendix*, Table S1). Indicative of an increased functional coupling between the VSD and the PD, the slope of voltage dependence of activation was significantly reduced (Fig. 4 *A* and *B* and *SI Appendix*, Table S1). Moreover, a higher fraction of F1362A channels remain noninactivating (Fig. 4 *A* and *B* and *SI Appendix*, Table S1), and mutant channels show slower inactivation kinetics after 50, 100, 250, and 500 ms (Fig. 4*C* and *SI Appendix*, Table S2) but faster deactivation kinetics at repolarization to −49.3 mV (*SI Appendix*, Fig. S2).

**Fig. 5.**
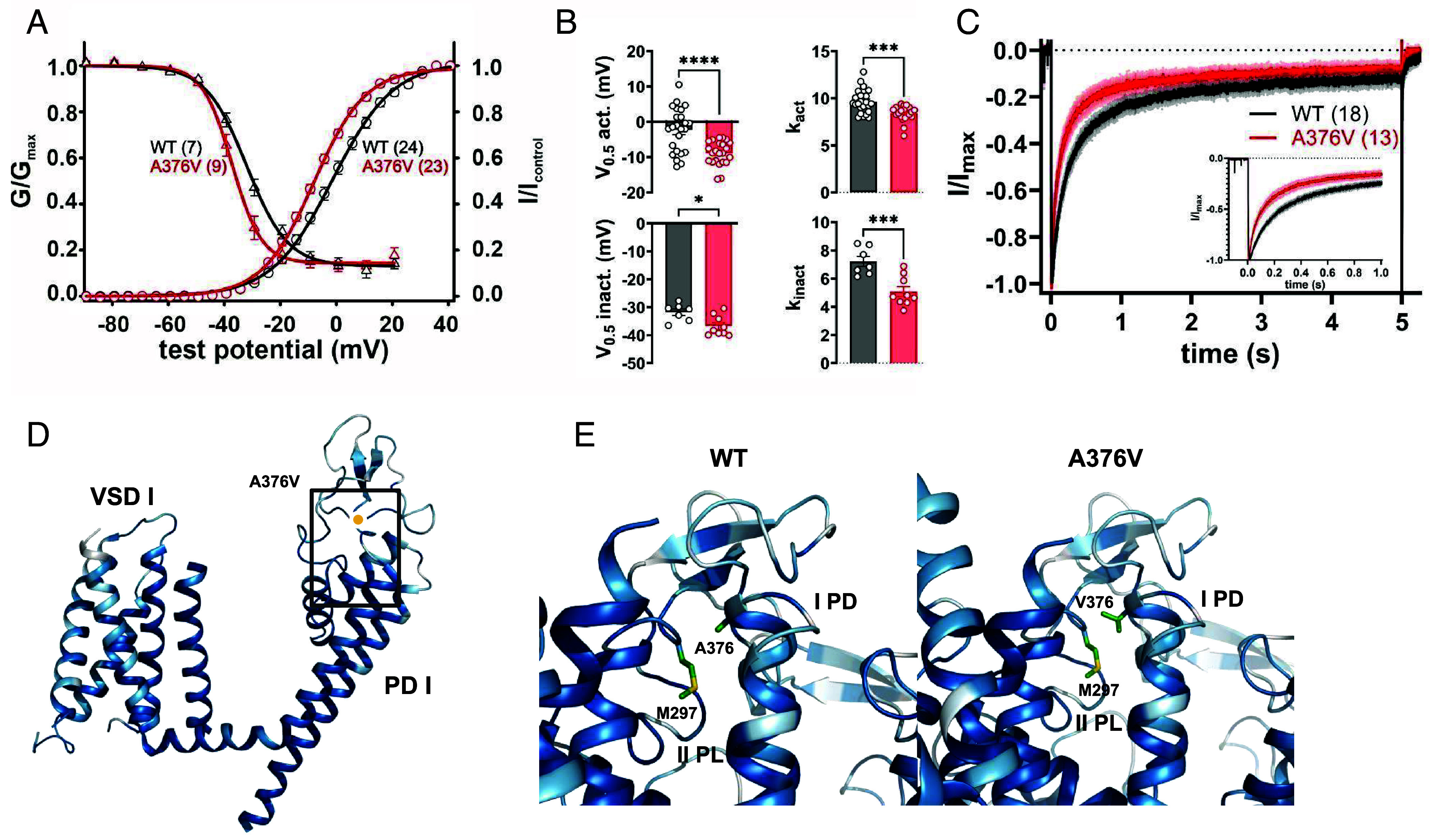
Structural and functional consequences of the A376V variant (domain I, pore loop). (*A*–*C*) The A376V induces a leftward shift in the voltage-dependent activation (WT: n = 24; A376V: n = 23) and steady-state inactivation (WT: n = 7; A376V: n = 9) along with accelerated inactivation (WT: n = 18; A376V: n = 13). (*D*) marks the position of A376V near the pore loop (PL) and the interaction network between PLs and those connecting PL to the rest of the pore domain (PD) in domain I. The structural illustration of the mutation effect in (*E*) shows that A376V replaces the minimal side chain of alanine with a much larger hydrophobic valine. This improves the PL–PD binding by strengthening the hydrophobic contact with M297 in II PL, while inducing distortion of the PL from its native conducting conformation. The side chains are colored according to the type of residues shown in Fig. 1.

The A376 is located in the PD of domain I (Fig. 5*D*). The substitution of alanine 376 with valine (A376V) introduces a much larger hydrophobic sidechain that strengthens the hydrophobic contact with M297 (Fig. 5*E*) improving the pore–loop–PD interaction. In contrast to the previously assumed loss-of-function, the A376V channel conducts calcium currents activated at ~−6 mV lower membrane potentials and with a steeper slope of voltage dependence of activation (Fig. 5 *A* and *B* and *SI Appendix*, Table S1). Current densities were significantly increased despite reduced apparent surface channel expression indicated by smaller Q_ON_ (*SI Appendix*, Fig. S1 and Table S1). Inactivation kinetics during the first 500 ms were significantly faster (*SI Appendix*, Table S2), while deactivation kinetics were unchanged (*SI Appendix*, Fig. S2).

**Fig. 6.**
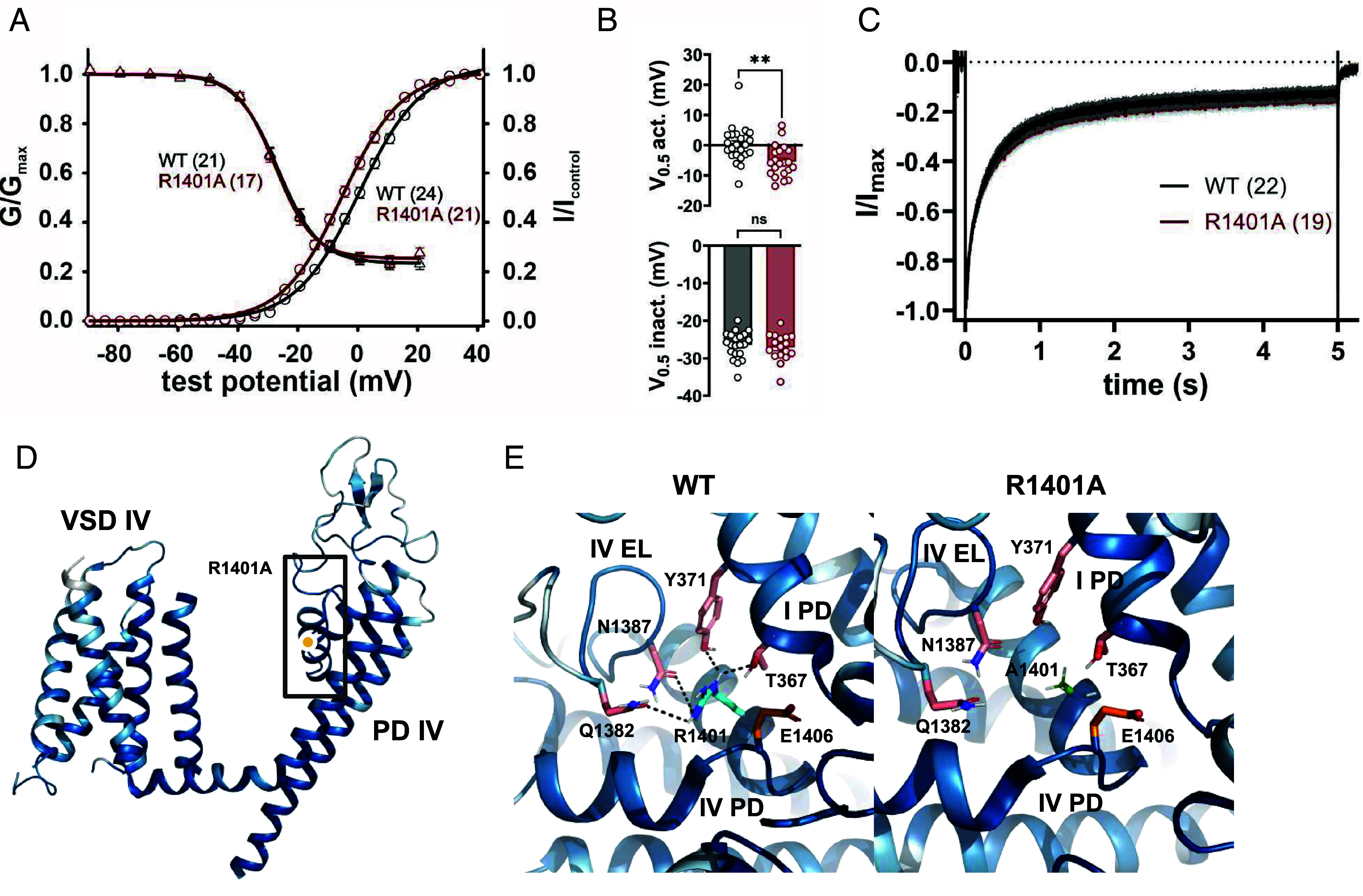
Structural and functional consequences of the R1401A variant (domain IV, pore loop). (*A*–*C*) R1401A induces a leftward shift in the voltage-dependent activation (WT: n = 24; R1401A: n = 21). (*D*) marks the position of R1401A in the interface between pore loops (PLs), near the selectivity filter and connecting the extracellular loop in domain IV. The structural illustration of the mutation effect in (*E*) shows that R1401A alters multiple polar contacts between PLs and to the extracellular loop. The side chains are colored according to the type of residues shown in Fig. 1.

Another pore loop variant, R1401A (domain IV), is located at the interface between pore loops near the selectivity filter (Fig. 6*D*). The alanine substitution alters multiple polar contacts between pore loops and to the extracellular S5–S6 loop (Fig. 6*E*), resulting in a significant shift in the voltage dependence of activation by ~−5.4 mV to more negative membrane potentials (Fig. 6 *A* and *B*) without altering the time course of inactivation (Fig. 6*C*) or any other gating parameters (*SI Appendix*, Figs. S1 and S2 and Tables S2).

**Fig. 7.**
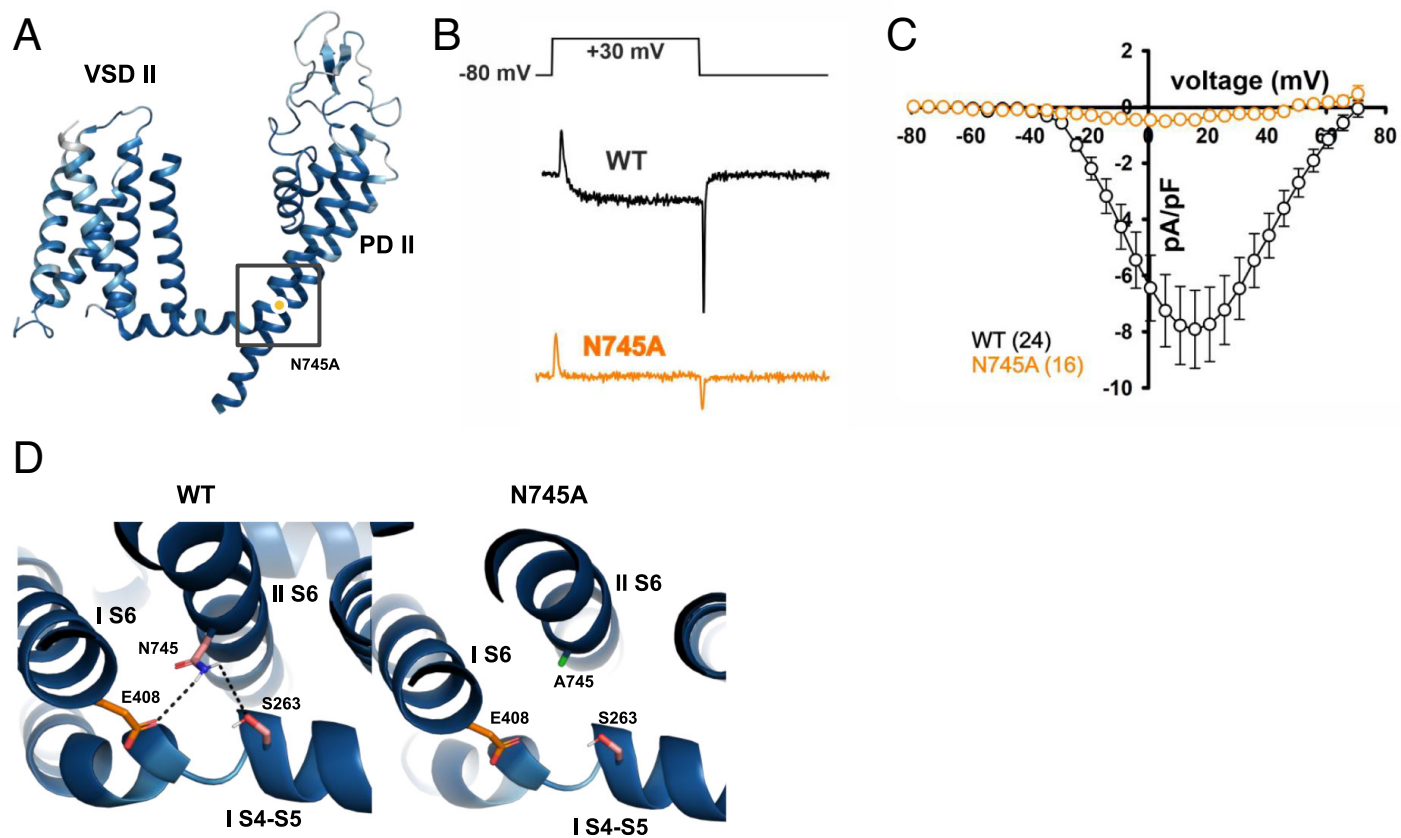
Structural and functional consequences of the N745A variant (domain II, S6). (*A*) marks the position of N745A and the interaction network surrounding the AG region detailed in *D*). (*B* and *C*) The N745A results in a nonconducting channel (WT: n = 24; N745A: n = 16). (*D*) provides structural illustration of the mutation effect that shows that N745A disrupts several contacts in the preopen state. The side chains are colored according to the type of residues shown in Fig. 1.

Near the functionally important LAIA motif within the activation gate of S6 in domain II (Figs. 1*F* and 7*A*), exchange of the highly conserved N745 residue to alanine disrupts several H-bonds that occur in the preopen state (Fig. 7*D*). While gating current measurements (Fig. 7*B* and *SI Appendix*, Table S1) demonstrate that N745A channels are expressed at the plasma membrane and respond to membrane depolarization, the altered stabilization of the activation gate completely abolishes the calcium influx (Fig. 7 *B* and *C*).

**Fig. 8.**
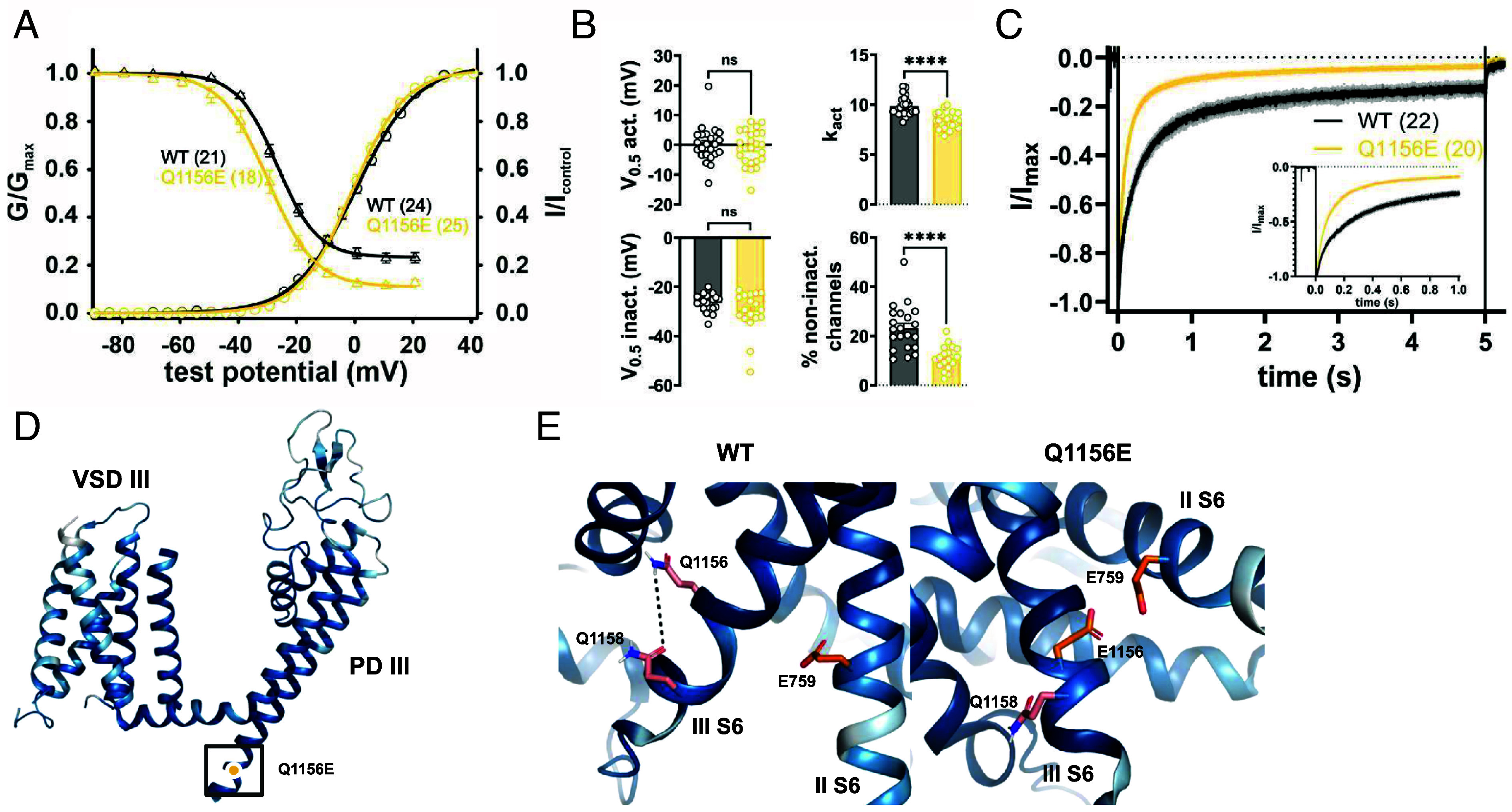
Structural and functional consequences of the Q1156E variant (domain III, S6). (*A*–*C*) The Q1156E enhances inactivation (WT: n = 22; Q1156E: n = 20) and reduces the number of noninactivated channels (WT: n = 21; Q1156E: n = 18). (*D*) marks the position of Q1156E in the channel structure (domain III). The structural illustration of the mutation effect in (*E*) shows that Q1156E substitutes an optimal hydrogen bond with electrostatic repulsion from E759. This change helps to move the distal III S6 into the ion path during inactivation. The side chains are colored according to the type of residues shown in Fig. 1.

Finally, at the cytoplasmic end of S6 in domain III (Fig. 8*D*), in wild type channels the glutamine Q1156 residue forms an interaction with glutamine Q1158 stabilizing the helix (Fig. 8 *E*, *Left*). Mutation to glutamic acid (Q1156E) did not shift the voltage dependence of gating, however, the voltage-sensitivity of channel activation was significantly increased (steeper activation slope factor; Fig. 8 *A* and *B* and *SI Appendix*, Table S1). The variant also increased current density (*SI Appendix*, Fig. S1 and Table S1) and the speed of inactivation (Fig. 8*C* and *SI Appendix*, Table S2), as well as increased total inactivation (Fig. 8 *A* and *B* and *SI Appendix*, Table S1). Structurally, the lack of interaction between Q1156 and Q1158 or increased electrostatic repulsion between E1156 and E759 (Fig. 8*E*) could reduce the stability of S6 facilitating a larger current and faster inactivation.

Together, these functional and structural data demonstrate that the purely evolutionary analysis of the Ca_v_1.3 pore-forming α1-subunit can indeed predict a functional impact of a distinct amino acid exchange on the channel function. Intriguingly, the model captured variants resulting in a large variety of gating changes, which affected different gating parameters (in various combinations and extents) that range from completely nonconducting channels to different levels of gain- and/or loss-of-function.

## Discussion

This study demonstrates the tremendous power of an evolution-based model (14) to uncover functional channel sites at individual amino acid level. Importantly, the evolutionary model is not used as a simple filtering step prior to structural analysis. Rather, it provides an orthogonal and predictive approach that enables prospective identification of functionally relevant residues, including sites outside classical functional regions, which would be difficult to anticipate based on structural considerations alone. Structural modeling, in contrast, is applied subsequently to rationalize the mechanistic basis of experimentally observed functional changes. Besides known key residues required for proper channel function, our model captured numerous sites along the Ca_v_1.3 α1-subunit with high context-dependent conservation and functional relevance. These evolutionarily conserved sites tend to form interconnected networks linked to known functional sites or cluster around previously identified functional residues. Structurally, the newly identified residues coordinate the conformational changes during state transitions therefore affecting channel biophysical properties.

As an orthogonal approach to clinical annotations and electrophysiological experiments, our evolutionary model demonstrated strong prediction validity and effectively identified residues that induce significant gating changes upon mutation. However, further analysis of different substitutions at the same position is at the moment essential to interpret the pathogenic potential of a residue. A key limitation of the current model is that it does not directly predict whether a given variant will result in gain- or loss-of-function. Rather, it identifies residues with a high likelihood of functional perturbation. In the case of *CACNA1D*, the direction of functional change can often be inferred from genetic context and clinical phenotype. For example, homozygous variants associated with sinoatrial node dysfunction and deafness (SANDD) syndrome typically reflect loss-of-function, whereas heterozygous variants linked to neurodevelopmental and endocrine phenotypes are generally associated with gain-of-function. Ultimately, electrophysiological characterization remains essential to define the precise functional consequences.

The model cannot faithfully predict the pathogenic potential of residues that have been highly conserved during evolution. Nevertheless, as more sequences from diverse species and individuals become available, the predictive value of the model will increase. Additionally, analysis of sequences from more distant voltage gated ion channel families may improve the prediction on positions with extremely low mutation rates since the functional properties remain conserved in the ion channel superfamily. Thus, the selection of the underlying sequence ensemble is a critical step for using evolutionary models. It is also important to consider sequence coverage and alignment confidence when interpreting results. Positions with low percentages of aligned sequences (e.g., below 50%) have lower confidence, whereas those with higher coverage (e.g., 80% or more) are generally reliable (14).

Interestingly, here we identified residues when mutated caused a broad range of gating changes that can lead to both channel loss- and gain-of-function features. Although ON gating current (Q_ON_) measurements suggested altered membrane expression for some, direct biochemical assays of surface expression will be needed for confirmation. In our previous study, we focused on de novo (i.e., heterozygous) variants that induce complex gating changes, which are predicted to overall enhance calcium influx and are associated with neurodevelopmental and endocrine dysfunction (14). However, here we also identified residues that when mutated cause a complete (N745A) or partial loss-of-function (e.g., F1362A). The N745A channel does not conduct an ionic current despite being expressed at the plasma membrane (visible Q_ON/OFF_ gating currents) while the F1362A mutant displays a significantly right-shifted voltage dependence of activation that most probably abolishes the channel’s native role as a pace-maker (43). Contrary, the shift of the voltage-dependent inactivation to more positive potentials will result in a higher fraction of available channels at physiologically relevant potentials compared to the WT, highlighting the complexity of mutation-induced channel dysfunction. Clinically, the complete (i.e., homozygous) Ca_v_1.3 loss-of-function causes the so-called SANDD syndrome, characterized by sinoatrial node dysfunction and deafness (44). The only SANDD variant functionally characterized to date, G403 duplication (44), produces the same effects on channel function as the N745A mutant described here. While Ca_v_1.3 loss-of-function mutations are likely to be asymptomatic in the heterozygous state (44), evolutionarily, a partial reduction in hearing acuity or impaired cardiac function would be highly detrimental. Therefore, our evolutionary based model is more capable of detecting channel functional sites compared to patient-identified mutations. However, in regard to clinical diagnostics, it poses the risk of “false-positive” predictions, underscoring the need for thorough functional testing.

Surprisingly, also the A376V mutation had been linked to SANDD syndrome-like auditory and cardiac deficiency (41, 42). However, our functional characterization demonstrates that A376V channels conduct robust calcium currents that display complex changes of channel properties similar to variants found in patients with neurodevelopmental disorders (15). One hypothesis could be that the patients with the inherited A376V variant bear other genetic mutation(s) that modify the observed clinical phenotype, including the lack of symptoms typically associated with enhanced Ca_v_1.3 activity. Alternatively, our simplified cell system might fail to detect complex channel alterations occurring in a native system. While heterologous overexpression in HEK cells allows a fast and easy biophysical characterization of isolated calcium current through the different channel variants, it does not take into account additional factors such as splicing events, RNA editing, post translational modifications, or protein–protein interactions, which would require more physiological model systems. While not all variants analyzed here are directly linked to patient cohorts, this framework is designed to enable rapid functional interpretation of newly identified *CACNA1D* variants in a clinical context.

In summary, the combination of thorough genetic testing, clinical information, functional characterization, and structural modeling still represents the gold standard for a reliable evaluation of pathogenic *CACNA1D* variants. Taken together, evolutionary and structural modeling address distinct but complementary questions: the former predicts which residues are functionally critical, while the latter explains how specific mutations alter channel behavior. This combination represents a generalizable framework for the study of ion channel variants.

## Conclusion

Here we demonstrated the potential of combining evolutionary, structural, and biophysical data to identify novel VGCC functional sites and to assess the pathogenicity of newly identified patient variants. This approach reliably uncovered clusters of residues with to date unknown functional impact, thereby setting the basis for future studies of novel intramolecular regulatory sites. While our study focused on Ca_v_1.3 channels, this approach can be prototypical for other ion channels, as they share high sequence homologies and gating mechanisms. By enabling prospective identification of functionally critical residues independent of prior annotation, this approach provides a scalable strategy for interpreting the rapidly increasing number of ion channel variants emerging from genomic studies.

## Materials and Methods

### Structural Models of Different Channel States.

Structural models of different states were built upon cryo-EM structures of channels captured near corresponding states. The homology modeling from template structures was conducted with MOE (Molecular Operating Environment, 2022.02 Chemical Computing Group ULC, Montreal, QC, Canada). To ensure the right match between the model sequence and the template, we cut the Ca_v_1.3 sequence (uniprot accession: CAC1D_human) into four domains and aligned them individually. Additionally, we checked and corrected the alignments with known functional sites, such as the S4 helices, selectivity filter, and known residues near the activation gate. The initial homology models were minimized with the Amber10:EHT forcefield with periodic boundary conditions (AMBER 10, University of California, San Francisco, CA, 2008).

The resting state was based on Nav1.7 (45) (PDB accession code: 6N4I) and the open state on Nav1.4 (46) (PDB accession code: 5XSY). We chose templates for the other states by comparing V_max_ of the template channel to the voltage of cryoEM experiments, i.e., 0 mV. This resulted in models of the preopen state based on Cav1.2 (47) (PDB accession code: 8WE6) and post-open on Cav1.1 (48) (PDB accession code: 5GJW). For additional quality control of the states modeled, we verified that models that are based on the more distant Cav2.2 (49) (PDB accession code: 7MIY) for preopen state and Ca_v_3.1 (40) (PDB accession code: 6KZO) for inactivated state yielded similar results. However, as residues are less similar and less well resolved for the models with these more distant channels, we only used the models basing on other L-type channels for the discussion.

### Evolutionary Model Building and Coupling.

The evolutionary prediction model originates from our recent publication (14), where the model building procedure and the quality control were described in detail. In the heatmap of the evolutionary model, each box represents a mutation whereas a column represents a target position marked by uniprot (accession: CAC1D_HUMAN) residue number. The WT scoring 0 was colored in white and marked with a dot. The darker the box, the more pathogenic the corresponding mutation was predicted to be.

### Visualization of Predicted Mutation Pathogenicity on Structural Models.

We employed the pymol package (PyMOL Molecular Graphics System, Version 2.0, Schrödinger, LLC) to map the pathogenicity of mutations onto the structural models as well as to visualize the structural impacts. By default, the cryoEM structure of Ca_v_1.3 (7) (PDB accession code: 7UHG) was chosen as template for plotting model predictions. The backbone was colored according to the most negatively scored mutation at a given position. To better distinguish the importance of residues predicted to be pathogenic to mutate, we set the cutoff to be colored white at the previously determined risk enhancing level of −4.6. Residues predicted to have more pathogenic mutations were colored in darker blue than those predicted to be more benign. Meanwhile, the sidechains were colored independently by residue type, e.g., polar not charged, hydrophobic and with different charges.

### Complementary DNA Constructs.

Human WT Ca_v_1.3 α1-subunit (full-length C-terminus, Ca_v_1.3_L_; GenBank accession number EU363339, but containing the alternative exon 8b: VNDAIGWEWPWVYFVSLIILGSFFVLNLVLGVLSG) was previously cloned into a pGFP^minus^ vector (no GFP tag, containing the CMV promoter) (21). Ca_v_1.3 missense variants A376V, S635I, N745A, Q1156E, R1401A, F1362A were obtained from GenScript. WT or mutant α1-subunits were coexpressed with auxiliary β3 (rat; GenBank accession number: NM012828) and α2δ-1 (rabbit; GenBank accession number NM001082276). EGFP was cotransfected and used as transfection marker.

### Cell Culture and Transfection.

tsA-201 cells (subclone of HEK-293 cells stably expressing the SV40 temperature-sensitive T-antigen, ECACC, 96121229) were cultured as previously described (19). In brief, cells were cultured in Dulbecco’s modified Eagle’s medium (DMEM; Sigma-Aldrich, D6546), completed with 10% FBS (Gibco, 10270-106), 2 mM L-glutamine (Gibco, 25030-032), 10 U/ml penicillin (Sigma-Aldrich, P3032), and 10 μg/mL streptomycin (Sigma-Aldrich, S6501) at 37 °C and 5% CO_2_ in a humidified incubator. Cells were transiently transfected with 3 μg cDNA of WT or mutated α1-subunit, 2 μg of β3 subunit and 2.5 μg of α2δ-1 subunit using the Ca^2+^-phosphate coprecipitation method, always including 1.5 μg of EGFP. All data were obtained from at least three transfections with independent cDNA preparations. On the following day, cells were trypsinized (0.05% trypsin) and plated onto 35-mm culture dishes precoated with poly-L-lysine (Cat# P-2636; Merck KGaA, Darmstadt, Germany). Following replating, cells were kept at 30 °C and 5% CO_2_ and were subjected to electrophysiological recordings 48 to 80 h after transfection.

### Electrophysiological Recordings in tsA-201 Cells.

Whole-cell patch-clamp recordings in the voltage-clamp configuration were performed using borosilicate glass micropipettes (cat# 64-0792, Warner Instruments, Hamden, CT) with a resistance of 1.5 to 4.5 MΩ, pulled using a P-1000 Flaming/Brown micropipette puller (Sutter Instrument, Novato, CA) and fire-polished using a MF-830 microforge (Narishige Co, Tokyo, Japan). The recording solutions contained, in mM: extracellular: 15 CaCl_2_, 150 Choline-Cl, 1 MgCl_2_, 10 HEPES; intracellular: 135 CsCl, 10 Cs-EGTA, 1 MgCl_2_, 10 HEPES, 4 Na_2_-ATP. Solutions were adjusted to pH = 7.3 using CsOH. Recordings were performed at room temperature using an Axopatch 200B amplifier (Axon Instruments), digitized at 50 KHz using a Digidata 1,322A digitizer (Molecular Devices, San José, CA), low-pass filtered at 2 kHz, compensated for 60 to 90% of the series resistance and analyzed using the pClamp 10.7 software (Molecular Devices, San José, CA). Leak subtraction was performed online using a P/4 protocol (I-V relationship protocol, tail current protocol) or offline (inactivation kinetics upon 5 s depolarizing stimulus, steady-state inactivation protocol). All voltages were corrected for a liquid junction potential of −9.3 mV. The holding potential (HP) was set to −89 mV unless otherwise stated.

Current-voltage (I-V) relationships were obtained by 25 or 50 ms depolarizing square pulses from the HP to various test potentials in 5 mV increments and data were fitted using the following equation:

$$I=G_{\max}\times (V-V_{\mathrm{rev}})/(1+\text{exp}\left(\frac{-\left(V-V_{0.5act}\right)}{k_{\mathrm{act}}}\right)),$$

where *V_rev_* is the extrapolated reversal potential, *V* is the test potential, *G_max_* is the maximal conductance, *V_0.5 act_* is the voltage of half-maximal activation and *k_act_* is the slope factor.

The voltage dependence of Ca^2+^ conductance was fitted in accordance to a Boltzmann relationship:

$$G=G_{\max}/(1+\text{exp}\left(\frac{-\left(V-V_{0.5act}\right)}{k_{\mathrm{act}}}\right)).$$

Inactivation kinetics were determined by applying a 5 s long depolarizing pulse to *V_max_* (voltage of maximal activation) from HP and measured at predefined time points (50, 100, 250, 500, 1,000, and 5,000 ms) as the remaining current expressed as % of the peak inward current.

Steady-state inactivation was determined by calculating the ratio of 2 current amplitudes (*I/I_control_*) elicited by 25 or 50 ms test pulses to *V_max_*, separated by 5 s conditioning steps to various potentials (10 mV increments, 30 s between sweep starts, from HP). Curves were fitted using the following modified Boltzmann equation:

$$I=I_{\mathrm{ni}}+(1-I_{\mathrm{ni}})/(1+\text{exp}\left(\frac{V-V_{0.5inact}}{k_{\mathrm{inact}}}\right)),$$

where *I_ni_* is the noninactivating current component, *V* is the membrane potential, *V_0.5 inact_* is the voltage of half-maximal inactivation and *k_inact_* is the slope factor.

Deactivation kinetics (tail currents) were determined from a 10 ms depolarizing step to *V_rev_* followed by 20 ms repolarizations to various test potentials (10 mV increments). Traces at a certain potential were normalized to the peak of the respective tail current during the repolarizing step, and the area under the curve (AUC) during the repolarization of each sweep was integrated in Clampfit.

### Statistical Analysis.

Electrophysiological data were analyzed using Clampfit 10.7 (Axon Instruments), Microsoft Excel, SigmaPlot 14.5 (Systat Software, Inc), GraphPad Prism 10 (GraphPad software, Inc), and MATLAB R2022b (MathWorks, Inc). Data are presented as means ± SEM for the indicated number of experiments (*n*) unless otherwise stated. Data were tested for Gaussian distribution using the Shapiro–Wilk test. Data were analyzed using unpaired Student’s *t* test, Mann–Whitney test, or two-way ANOVA followed by Šidák’s multiple comparisons test. Differences were considered statistically significant when *P < 0.05, **P < 0.01, ***P < 0.001 and ****P < 0.0001.

## Supplementary Material

Appendix 01 (PDF)

Dataset S01 (XLSX)

Dataset S02 (XLSX)

## Data Availability

All study data are included in the article and/or supporting information.
