# Supplementary material for: Identification of novel functional sites in the Cav1.3 calcium channel α1-subunit using evolutionary modeling
Source: Proc Natl Acad Sci U S A. 2026 Aug 3;123(32):e2602636123. doi: 10.1073/pnas.2602636123 (PMC13462451; doi:10.1073/pnas.2602636123)
Supplement: Supplementary file 1 [file pnas.2602636123.sapp.pdf]

Supporting Information for

Identification of novel functional sites in the Cav1.3 calcium channel α1 subunit using evolutionary modeling

Authors:

Xuechen Tang[1,†], Horia C. Hermenean[2,†], Alesia Yakimchyk, Petronel Tuluc[2], Nadine J. Ortner[2,*], Klaus R. Liedl[1,*]

* Corresponding authors: Klaus R. Liedl, Faculty of Chemistry and Pharmacy, University of Innsbruck, Innrain 82, A-6020 Innsbruck, Austria, +43 512 50757100, Klaus.Liedl@uibk.ac.at; Nadine J. Ortner, Medical University Graz, Neue Stiftingtalstraße 6/III, A-8010 Graz, Austria, +43 316 385 71695, Nadine.Ortner@uibk.ac.at.

This PDF file includes:

Figures S1 to S2

Tables S1 to S2

Figures

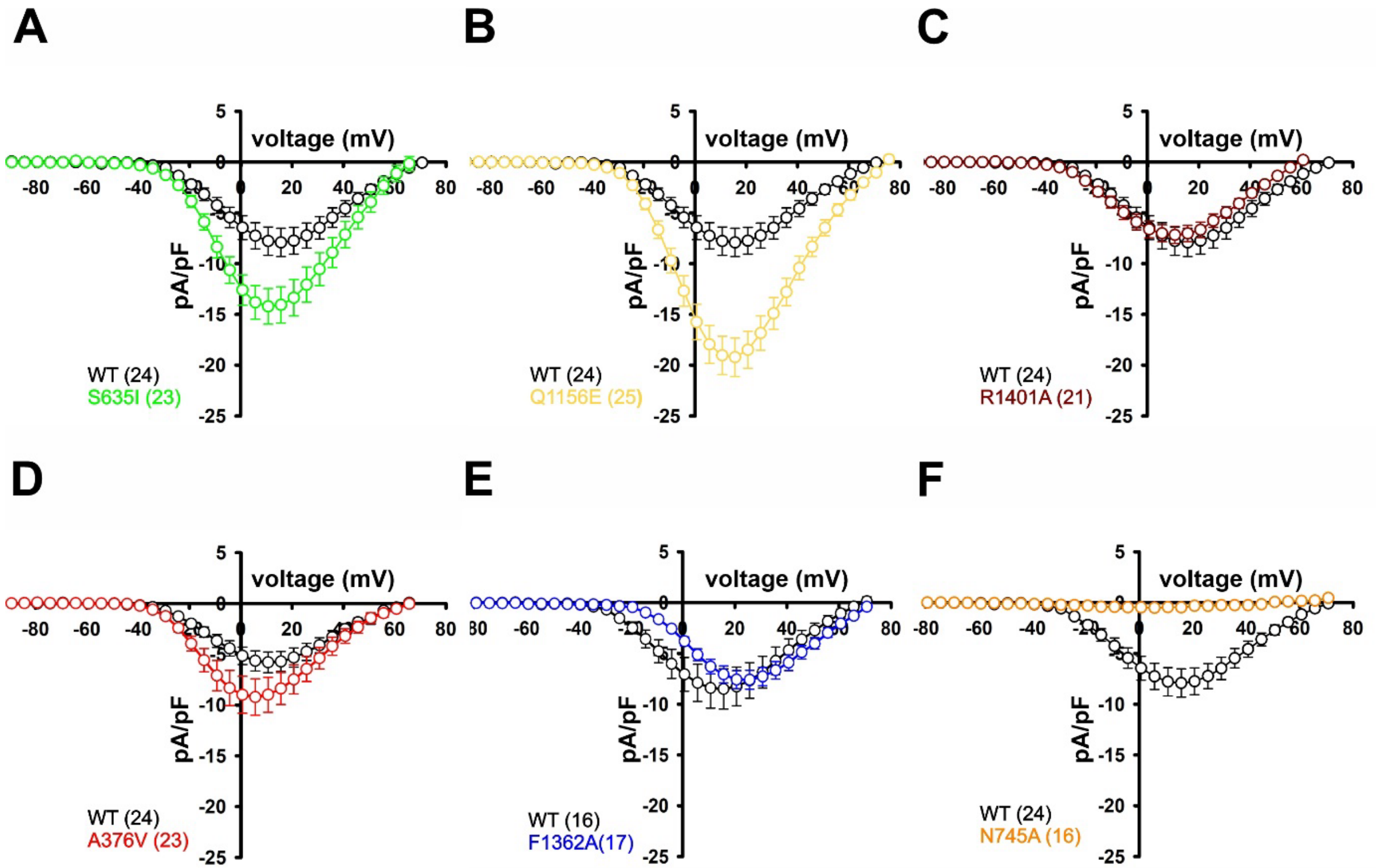

Fig. S1. Current density plots of all tested Cav1.3 variants.
Current-voltage relationships (I-V) values normalized to the cell capacitance. Statistics were performed between the current densities of WT and mutant channels at the voltage of maximal activation (V_{max}) of each individual cell. The variants S635I (**A**), Q1156E (**B**) and A376V (**D**) significantly increased the current density (Mann-Whitney test, *** $p<0.001$ for S635I; **** $p<0.0001$ for Q1156E; ** $p<0.01$ for A376V). Variants R1401A (**C**) and F1362A (**E**) did not alter the current amplitudes of Cav1.3 channels. Lastly, the N745A variant (**F**) led to a nonconducting channel without detectable inward calcium currents.

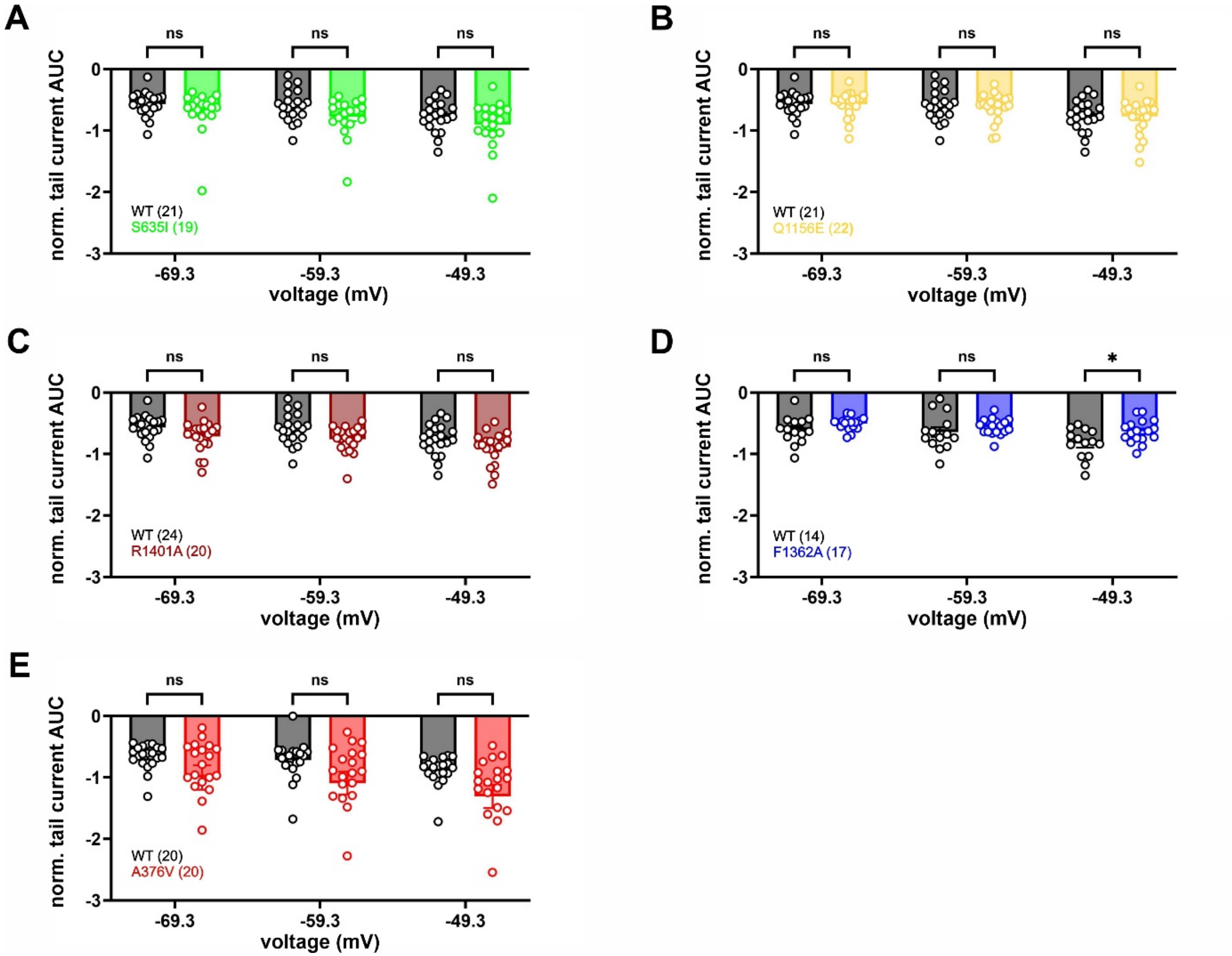

Fig. S2. Deactivation (tail current) kinetics of Cav1.3 WT and mutant channels.
Normalised tail current area under the curve (AUC) was calculated as detailed in Methods. The F1362A variant (**D**) was the only variant from this study found to induce an effect on deactivation kinetics, i.e. significantly faster deactivation kinetics compared to WT at -49.3 mV (2-way ANOVA: voltage factor ** $p<0.01$; genotype factor ** $p<0.01$; Šidák's multiple comparisons test: * $p<0.05$ at -49.3 mV).

Tables

Table S1. Steady-state activation and inactivation parameters of Cav1.3 wildtype (WT) and mutant channels.

α1 subunit	Activation								Steady-State Inactivation			
	$V_{0.5\ act}$ (mV)	k_{act}	V_{rev} (mV)	act. thres. (mV)	Current density (pA/pF)	n	Q_{ON} (pA*ms)	n	$V_{0.5\ inact}$ (mV)	k_{inact}	non-inact. (%)	n
**WT**	0.27 ± 1.22	9.85 ± 0.19	65.3 ± 1.56	-38.5 ± 0.73	-8.04 ± 1.38	24	66.3 ± 11.9	24	-26.4 ± 0.78	6.69 ± 0.35	23.2 ± 2.04	21
**S635I**	-4.22 ± 1.02 **	8.18 ± 0.2 ****	61.3 ± 1.89	-35.1 ± 1.2	-15.7 ± 1.88 ***	23	129.2 ± 25.0 *	23	-28.6 ± 1.22	6.73 ± 0.2	14.5 ± 2.47 **	16
**R1401A**	-5.38 ± 1.14 **	9.35 ± 0.21	61.23 ± 1.41	-41.5 ± 0.57	-7.35 ± 0.9	21	77.4 ± 14.4	21	-27.1 ± 0.88	6.22 ± 0.25	24.7 ± 2.41	17
**Q1156E**	-1.0 ± 1.15	8.43 ± 0.15 ****	62.1 ± 1.3 ***	-33.1 ± 1.1	-20.0 ± 2.0 ****	25	114.7 ± 26.6	24	-30.2 ± 2.04	6.47 ± 0.22	11.2 ± 1.14 ****	18
**WT (F1362A)**	-0.91 ± 1.23	9.88 ± 0.24	64.4 ± 1.28	-39.6 ± 0.97	-8.63 ± 1.98	16	77.2 ± 16.8	16	-26.8 ± 1.2	6.54 ± 0.43	21.6 ± 2.43	13
**F1362A**	9.8 ± 1.12 ****	8.87 ± 0.23 **	70.2 ± 1.33 **	-24.7 ± 0.84 ****	-7.83 ± 0.92	17	98.8 ± 16.1	17	-11.4 ± 1.08 ****	6.84 ± 0.46	32.7 ± 3.98 *	12
**WT (A376V)**	-2.31 ± 1.28	9.66 ± 0.25	57.9 ± 1.57	-40.3 ± 0.86	-5.98 ± 1.01	24	96.5 ± 17.0	20	-31.8 ± 1.16	7.22 ± 0.37	12.2 ± 2.78	7
**A376V**	-8.84 ± 0.7 ****	8.4 ± 0.16 ***	56.7 ± 1.2	-40.7 ± 0.72	-9.52 ± 1.78 **	23	49.5 ± 12.8 **	23	-36.7 ± 1.2 *	5.08 ± 0.35 ***	13.5 ± 2.38	9

Gating parameters were obtained by fitting the current-voltage relationships for activation (G/G_{max}) and steady-state inactivation (I/I_{max}) to Boltzmann functions, as described in Methods. Data are presented as mean ± SEM from at least 3 independent transfections and the number of recordings from individual cells is indicated (*n*). Data were analysed using the unpaired Student's t-test or the Mann-Whitney test and differences were considered statistically significant when * $p<0.05$, ** $p<0.01$, *** $p<0.001$ and **** $p<0.0001$. Statistical significances are given for each variant in comparison to WT data measured within the same transfection, therefore WT data can differ between variants. $V_{0.5,act}$, voltage of half-maximal activation; k_{act}, activation slope factor; V_{rev}, extrapolated reversal potential; current density, current amplitude normalized to the capacitance (i.e. cell size); Q_{ON}, on-gating currents; $V_{0.5,inact}$, voltage of half-maximal inactivation; k_{inact}, inactivation slope factor; non-inact., non-inactivating component of the steady-state inactivation curve.

Table S2: Inactivation kinetics time course of Cav1.3 WT and mutant channels.

α1 subunit	r50	r100	r250	r500	r1000	r5000	n
**WT**	0.78 ± 0.02	0.67 ± 0.02	0.5 ± 0.02	0.35 ± 0.02	0.24 ± 0.02	0.13 ± 0.02	22
**S635I**	0.83 ± 0.02	0.69 ± 0.03	0.46 ± 0.03	0.3 ± 0.03	0.19 ± 0.02	0.07 ± 0.01	18
**R1401A**	0.78 ± 0.02	0.66 ± 0.02	0.5 ± 0.02	0.37 ± 0.02	0.27 ± 0.02	0.15 ± 0.02	19
**Q1156E**	0.62 ± 0.03 ****	0.43 ± 0.03 ****	0.23 ± 0.02 ****	0.14 ± 0.02 ****	0.09 ± 0.01 ****	0.04 ± 0.01 *	20
**WT (F1362A)**	0.79 ± 0.03	0.68 ± 0.03	0.51 ± 0.03	0.37 ± 0.03	0.26 ± 0.03	0.14 ± 0.03	16
**F1362A**	0.94 ± 0.02 ***	0.82 ± 0.02 **	0.63 ± 0.02 *	0.47 ± 0.03 *	0.33 ± 0.03	0.16 ± 0.03	16
**WT (A376V)**	0.85 ± 0.02	0.71 ± 0.03	0.5 ± 0.03	0.35 ± 0.03	0.24 ± 0.03	0.12 ± 0.02	18
**A376V**	0.65 ± 0.05 ***	0.51 ± 0.05 ***	0.33 ± 0.05 **	0.22 ± 0.04 *	0.15 ± 0.03	0.08 ± 0.02	13

r values represent the remaining current ratio (between the calcium current measured at prespecified time points [50, 100, 250, 500, 1000, 5000 ms] and the normalised peak inward current I/I_{max}) during a 5 s depolarising step pulse to the voltage of maximal activation (V_{max}) from the holding potential (*HP*). Values are presented as mean ± SEM from the indicated number (*n*) of recordings, obtained from at least 3 independent transfections. Data were analysed using 2-way ANOVA followed by Šidák's multiple comparisons test and differences were considered statistically significant when * p<0.05, ** p<0.01, *** p<0.001 and **** p<0.0001. Statistical significances are given for each variant in comparison to WT data measured within the same transfection, therefore WT data can differ between variants.
